# Leveraging the subgenus category to address monophyletic genus over-splitting: illustration with recently proposed Mycobacteriales genera

**DOI:** 10.1099/ijsem.0.006917

**Published:** 2025-09-19

**Authors:** Jorge Val-Calvo, Mariela Scortti, Markus Göker, José A. Vazquez-Boland

**Affiliations:** 1 Microbial Pathogenomics Laboratory, Edinburgh Medical School (Biomedical Sciences) and Institute for Regeneration and Repair (IRR), Edinburgh BioQuarter, Edinburgh EH16 4UU, Scotland, UK; 2 Leibniz Institute DSMZ, German Collection of Microorganisms and Cell Cultures, 38124 Braunschweig, Germany

**Keywords:** monophyletic genus over-splitting, subgenus category, context-uniform taxon demarcation, network analysis-aided taxon demarcation, *Mycobacteriales *taxonomy, genus* Rhodococcus*, subgenus *Rhodococcus*, subgenus *Prescottella*, subgenus *Anisorhodococcus*, subgenus *Pararhodococcus*, genus* Rhodococcoides*, genus* Mycobacterium*, subgenus *Mycobacterium*, subgenus *Mycolicibacterium*, subgenus *Mycolicibacter*, subgenus *Mycolicibacillus*, genus *Mycobacteroides*

## Abstract

Three related circumstances are affecting the stability of prokaryotic taxonomy and nomenclature, with significant implications in the field of pathogenic micro-organisms: (i) the subjective application of genomics-based demarcation criteria to subdivide monophyletic genera, creating an increasing number of new genera; (ii) databases’ preference for the latest validly published names; and (iii) the practical irreversibility of new names in databases, even when later taxonomic opinion supports reverting to previous classifications. Due to understandable end-user reluctance to accept name changes affecting well-known pathogens, parallel nomenclatures coexist, causing confusion. To address this issue, we propose using the subgenus category to mitigate the disruptive impact of genus name changes in databases. Specifically, we suggest lowering to subgenus rank those new genera arising from monophyletic genera splits that have limited practical utility and may contribute to taxonomic instability. Under the International Code of Nomenclature of Prokaryotes, the species’ generic name would revert to its previous synonym, optionally followed in parentheses by the validly published subgenus name (corresponding to the latest genus synonym used in databases). Because the subgenus is an optional taxonomic category, it may be omitted; however, its use may facilitate the mapping of synonyms in databases and literature. We illustrate this strategy through its application to recent genus splits in the *Mycobacteriales*, specifically the genus *Prescottella* nested within the rhodococcal radiation, and the several genera into which *Mycobacterium* was subdivided.

A fundamental principle of prokaryotic nomenclature, alongside taxonomic freedom, is to aim at the stability of names [principle 1.1. of the International Code of Nomenclature of Prokaryotes (ICNP)] [1]. Stable nomenclature is essential for the effective study of microbiology, the communication of microbiological knowledge and the traceability of microbial species in the literature. This notion is particularly evident in human and veterinary medicine, clinical microbiology and public health, where microbial name changes, in addition to creating confusion, can lead to identification errors, misdiagnoses and inaccurate risk assessments. However, taxonomic and nomenclatural stability is increasingly compromised by the growing trend of subdividing existing genera into multiple new ones [2,10].

This practice, referred to here as ‘genus over-splitting’, is a paradoxical consequence of the subjective implementation of a (potentially objective) genomics-based taxonomy. For clarity, we define ‘genus over-splitting’ as any subdivision of monophyletic genera that lacks clear and robust practical justification and is potentially disruptive to taxonomic and nomenclatural stability. This phenomenon was recently analysed and documented in a taxonomic study of the order *Mycobacteriales* [11], affected by two significant examples of monophyletic genus fragmentation: the controversial [12,14] subdivision in 2018 of *Mycobacterium* into five genera [6], and the creation in 2022 of the nested genus *Prescottella* for the rhodococcal sublineage containing *Rhodococcus equi* [15]. The latter rendered the genus *Rhodococcus* paraphyletic, implying a need either to revert to the previous classification or to establish additional genera for each of the major rhodococcal sublineages, in order to ensure monophyly throughout. Using a novel, network analysis-aided approach for taxonomic rank demarcation, the *Mycobacteriales* study showed that the genus splits resulted from applying arbitrary genomic relatedness index (GRI, aka OGRI [16])-based boundaries that systematically elevated intra-generic sublineages to the genus level [11]. Specifically, the proposed novel *Prescottella* genus involved shifting the genome-aggregate average amino acid identity (AAI) demarcation threshold to 74–75% [15], significantly deviating from the proposed 65% standard for genus definition in both natural isolates and metagenomic sequences [17,20]. There was also inconsistent use of other GRIs, including values for the percentage of conserved proteins (POCP) far exceeding the proposed 50% threshold for genus demarcation [21].

Subdivision of established monophyletic genera is possible due to the lack of standardized genome-based demarcation guidelines, combined with the freedom of taxonomic thought (principle 1.4 of ICNP [1]). Upon valid publication, the resulting new names are immediately adopted by all major databases, notably the National Center for Biotechnology Information (NCBI)’s taxonomy browser and nucleotide sequence repository (GenBank). This information is automatically mirrored by other gene and genome databases, such as the European Nucleotide Archive at the European Bioinformatics Institute (EBI-EMBL) and the DNA Data Bank of Japan (DDBJ), within the framework of the International Nucleotide Sequence Database Collaboration (INSDC; http://www.insdc.org/) [22]. Indeed, one of the goals of the INSDC initiative is to use a unified taxonomy across all databases based on sequence information (https://ncbi.nlm.nih.gov/genbank/collab/) [23].

## Database preference for latest validly published names

Since the proposal of new combinations does not change the fact that the earlier names are validly published and legitimate according to ICNP rules, both the new and earlier names become synonyms [1]. Therefore, users are free to use the previous name (or any other validly published and legitimate earlier synonym) [1,24,26] if in disagreement with the taxonomic and nomenclatural changes. Ultimately, these changes reflect only the taxonomic opinion of those who proposed them. Additionally, the NCBI Taxonomy Project/Database states that it is ‘not an authoritative source for nomenclature or classification’ (https://www.ncbi.nlm.nih.gov/Taxonomy/Browser/wwwtax.cgi) [27,28]; only the ICNP and the International Committee on Systematics of Prokaryotes (ICSP) hold that prerogative (and only for nomenclature). However, in practice, the NCBI Taxonomy and mirror databases introduce a powerful ‘preference’ bias towards specific names/synonyms, in two ways.

First, because of NCBI’s dominant position as a trusted public biosciences information repository, the taxonomic names they use are potentially perceived as representing the ‘officially sanctioned’ name for a species. This perception is likely to influence the choice of database users, who may not be familiar with ICNP’s rules of synonymy. For the same reason, journal editors may also request authors to follow the ‘official’ nomenclature of the NCBI Taxonomy database. The NCBI itself explicitly recognizes the concept of ‘preferred name’ in its Taxonomy Browser (https://www.ncbi.nlm.nih.gov/books/NBK53758) [29], favouring particular combinations over other validly published and legitimate names (synonyms). For example, *Mycolicibacterium smegmatis* is indicated as the preferred name for *Mycobacterium smegmatis*, despite the 2021 emendation of mycobacterial nomenclature which reclassified the four additional genera created in 2018 [6] (see below) back into a single genus, *Mycobacterium* [14,30, 31]. Similarly, NCBI still considers *Prescottella equi* as the preferred name, rather than *R. equi* (see entry at https://www.ncbi.nlm.nih.gov/Taxonomy/TaxIdentifier/tax_identifier.cgi), despite a recent change in taxonomic opinion which reclassified the species back into the genus *Rhodococcus* [11].

Second, NCBI has adopted the policy of using the most recently proposed synonym that has obtained standing under ICNP rules as their ‘primary name’ [27] (i.e. the name chosen out of all synonyms as the designated label for the TaxNode and its TaxID numerical identifier, also referred to as ‘preferred name’; https://www.ncbi.nlm.nih.gov/Taxonomy/TaxIdentifier/tax_identifier.cgi). This policy is implemented despite the ICNP not stipulating that the most recent validly published and legitimate name should be treated as the correct name for a species over all its synonyms. Margos *et al*. [25] explain this practice, quoting the following statement from NCBI: ‘In the case of two validly published names, one being a new combination of an earlier name, priority is given in the NCBI taxonomy database to the latest validly published name’. This has two important practical consequences.

One is that it negates the possibility that changes in taxonomic opinion based on new evidence, better data or scientific advances – published as emendations and duly notified in the ‘Lists of Changes in Taxonomic Opinion’ [32] – are adequately reflected in the databases. As a result, any proposed new name becomes virtually irreversible, effectively undermining the freedom of taxonomic thought and action. The ‘preference for latest validly published name’ policy has a major negative impact in situations where further taxonomic research has concluded that reverting to an earlier classification is more appropriate. This applies particularly when subdivisions of well-established, monophyletic genera – leading to the creation of new generic names – appear to be unwarranted. Clear examples are the previously discussed case of the genus *Prescottella* nested within the *Rhodococcus* genus radiation, or the split of *Mycobacterium* into five genera [11,14].

The other is that, when new species are described using the earlier genus synonym rather than the latest validly published name adopted by databases, NCBI Taxonomy marks their taxonomic check status as ‘inconclusive’ (see, for example, https://www.ncbi.nlm.nih.gov/datasets/genome/GCF_963378085.1/, accessed 7 April 2025). Alternatively, it lists the species with the earlier genus name in square brackets, accompanied by the caveat ‘awaits appropriate action by the research community to be transferred to another genus’ – namely, the one associated with the latest validly published name (see, for example, https://www.ncbi.nlm.nih.gov/Taxonomy/Browser/wwwtax.cgi?mode=Info&id=3064284&lvl=3&lin=f, accessed 7 April 2025). In this way, NCBI appears to be calling for a publication formalizing a new combination that uses the most recent validly published genus name, even if that name goes against current taxonomic opinion. In other words, the ‘preference for latest validly published name’ policy, as applied by databases, not only appears to contravene the principle of freedom of taxonomic thought but may also unduly influence taxonomic action and the development of microbial taxonomic research.

## Use of the subgenus category to rectify splits of monophyletic genera

In cases where reconsideration of a genus split is advisable, one possible strategy to address database prioritization of the latest validly published names is to make use of the subgenus category – an available, although seldom used, taxonomic category under the ICNP [1]. Essentially, this approach involves lowering in rank those new genera arising from monophyletic genus splits that are deemed of limited value and are needlessly disruptive to taxonomic stability, thereby allowing for the valid publication of new subgenus names. Doing so should result in the reinstatement of the earlier (pre-split) binomial designation in databases, in application of their ‘preference for latest validly published name’ policy. According to ICNP rule 10c for subgenus notation [1], the species name may also optionally include, placed in parentheses before the epithet, the latest genus name (i.e. the one being replaced) along with the abbreviation subgen., thus facilitating the tracking and retrieval of the synonyms. This approach is illustrated below with the recently proposed nested rhodococcal genus *Prescottella* Sangal *et al*. 2018. For example, under the proposed action, the name *P. equi* would change to *Rhodococcus* (subgen. *Prescottella*) *equi* (or simply *R. equi*, the earlier synonym by which the species was known).

## Demarcation of rhodococcal subgenera

To accurately define the subgenus circumscriptions, we applied our recently reported phylogenomic approach for taxon demarcation, based on normalized tree clustering and network analysis of GRI and maximum likelihood (ML) distance (MLD) matrices [11]. Originally developed for genus delineation, this methodology has proven useful as a general taxonomic rank demarcation tool. To minimize demarcation subjectivity, the method involves the uniform application of the same tree clustering and network graph partitioning thresholds across a sufficiently broad ‘taxonomic context’ – not just the specific circumscription under study. For genus delineation, the taxonomic context was set at the order level, using the classical (pre-genomic) genera within that context as a demarcation reference to ensure taxonomic and nomenclatural continuity [11]. For *Rhodococcus* subgenus demarcation, we are using here the entire *Nocardiaceae* radiation as the taxonomic context, including the recently proposed families *Hoyosellaceae* and *Tomitellaceae*, which together form a major line of descent within the *Mycobacteriales* [11,33]. The analyses included representative genome sequences of the genera *Nocardia*, *Antrihabitans*, *Rhodocococcus*, *Rhodococcoides*, *Tomitella*, *Hoyosella* and *Lolliginicoccus* (the latter recently proposed within the family *Hoyosellaceae* [34]), as well as the monotypic genera *Aldersonia* and *Skermania* (Fig. S1 and Supplementary Dataset, available in the online Supplementary Material).

**Fig. 1.**
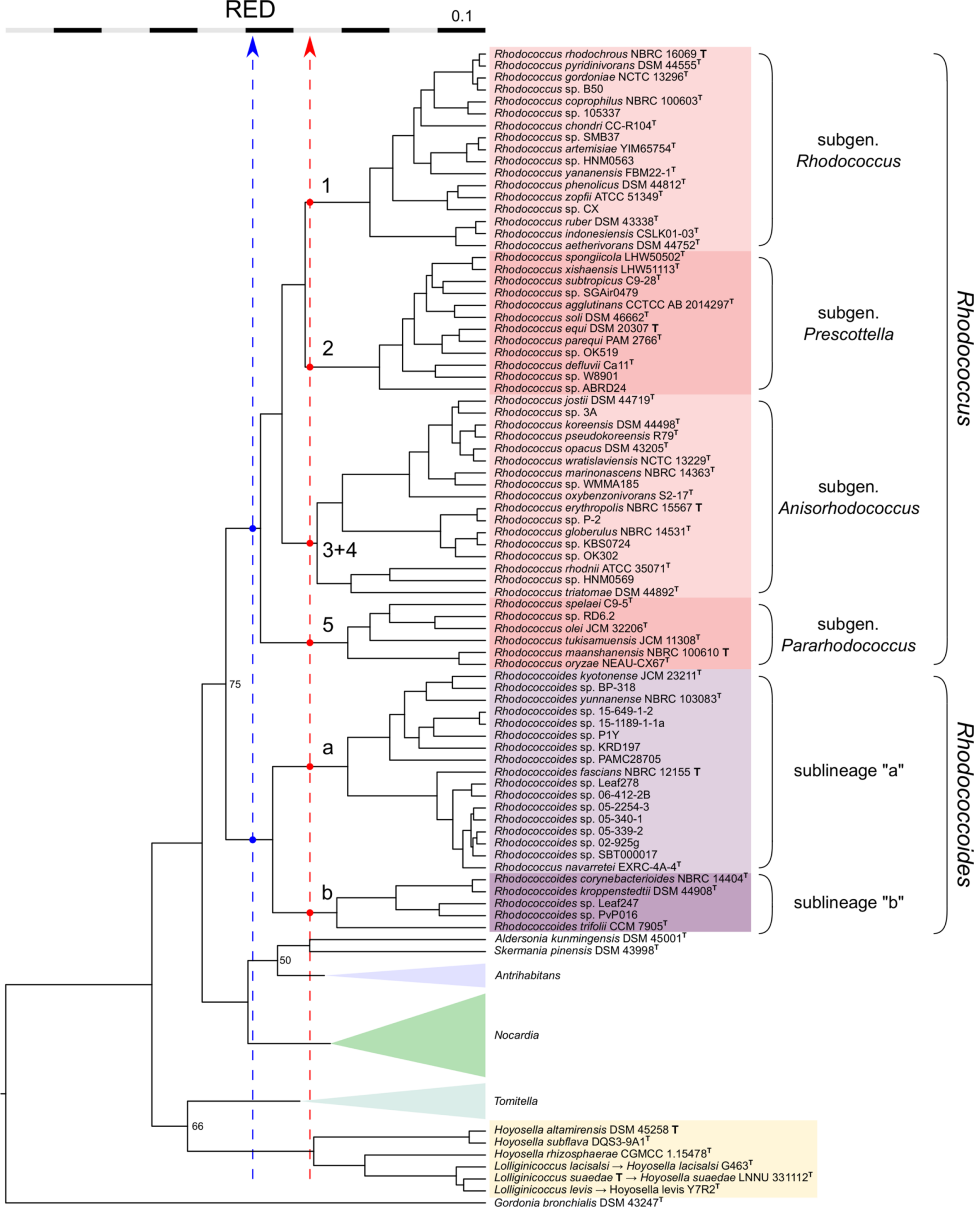
RED-normalized phylogenomic ML tree of the families *Nocardiaceae*, *Hoyosellaceae* and *Tomitellaceae*. See Fig. S1 for the non-normalized version of the tree and details of the phylogenetic analysis. The genera *Nocardia*, *Antrihabitans* and *Tomitella* are collapsed. Dashed lines represent TreeCluster partitioning thresholds used to define genera (blue) and subgenera (red). Dots mark where these thresholds intersect with the rhodococcal radiation. Major sublineages within the genera *Rhodococcus* (shaded red) and *Rhodococcoides* (shaded mauve) are labelled with numbers [45] and lowercase letters, respectively. Proposed subgenera are indicated on the right with braces. Note the following: (**i**) the genus-level partition does not support the proposed new genus *Lolliginicoccus* within the *Hoyosella* radiation; (ii) the recently described species *Rhodococcus navarretei* falls within sublineage ‘a’ of the genus *Rhodococcoides*. Bootstrap values below 75 (1,000 replicates) are shown. Tree was visualized using FigTree v1.4.4 (http://tree.bio.ed.ac.uk/software/figtree/). The genomes used for each of the listed species are from the type strains (indicated by superscript T, a bold T in normal font designates genus/subgenus type species); their accession numbers are provided in the Supplementary Dataset table.

We began by constructing a detailed ML phylogeny using a total of 175 genome sequences from the type strains of all the species with available sequences within the study’s taxonomic context. To achieve a more comprehensive representation of the diversity within the *Rhodococcus*/*Rhodococcoides* radiation, the dataset also included 31 unclassified *Rhodococcus* spp. For these, one genome was selected from each main terminal branching in the *Rhodococcus* spp. genomic blast dendrogram available at NCBI (https://www.ncbi.nlm.nih.gov/genome/?term=txid192944, accessed November 2022), using an average nucleotide identity (ANI) filter of ≥95% (the standard cutoff for species delineation [18,35,37]) (Fig. S1 and Supplementary Dataset). The TreeCluster program’s ‘Max Clade’ clustering method [38] was then used to partition the ML tree into discrete clusters based on evolutionary distances (branch lengths) and tree topology (phylogenetic relationships). To avoid clustering biases caused by differences in evolutionary rate across the tree, branch lengths were first normalized by relative evolutionary divergence (RED) using the PhyloRank package [39] (Fig. 1). For the taxonomic context under study, a TreeCluster threshold *t*=0.95 recapitulated the genus structure of the *Nocardiaceae* radiation [11]. Lowering the *t* value to 0.75 partitioned the tree into clusters that, within the rhodococcal radiation, roughly corresponded to the major intra-generic sublineages (Fig. 1).

These analyses yielded the following conclusions:

1. Four major monophyletic lines of descent (designated by Arabic numerals in Fig. 1) can be identified within the genus *Rhodococcus* (2023 emendation [11,40]) when using the ‘Prescottella’ sublineage (aka, sublineage no. 2 or ‘equi’ clade) as a partition reference. For internal taxonomic consistency, all four of these sublineages should be considered *Rhodococcus* subgenera if the *Prescottella* circumscription is classified under this taxonomic category.
2. Two sublineages hierarchically equivalent to those above (designated ‘a’ and ‘b’ in Fig. 1) are also observed in the genus *Rhodococcoides* [11] and, accordingly, could also be treated as subgenera.
3. Using the same genus and subgenus tree partitioning criteria, *Lolliginicoccus* [34] would not warrant independent genus status (it would not even qualify as a *Hoyosella* subgenus) (Fig. 1).
4. The strong homogeneity of the *Nocardia* genus [11] is confirmed, with all its main sublineages radiating at short genetic distances well below the subgenus demarcation threshold (Figs 1 and S1).

## Taxonomic network analysis

Next, the subgenus rank demarcations based on tree clustering were validated using network analysis of MLD and GRI matrices [11]. The MLD matrix was constructed using the phylogenetic tree dataset from Fig. S1. The GRI matrices were based on AAI [17,19, 20] and aligned fraction of orthologous genes (AF) scores [41,42]. In these analyses, the MLD and GRI pairwise comparison matrices were used to generate correlation matrices, which were then visualized three-dimensionally (3D) as a network graph to examine the taxonomic relationships [11]. The network graphs were generated using Graphia, an updated version of BioLayout employed in our previous *Mycobacteriales* study, offering improved compatibility, correlation analysis for high-dimensional matrices and graphical environment [43,44]. To explore the degree of relatedness among the species included in the analysis, network graphs with increasing fragmentation were generated across a gradient of correlation/clustering threshold (ct) values.

**Fig. 2.**
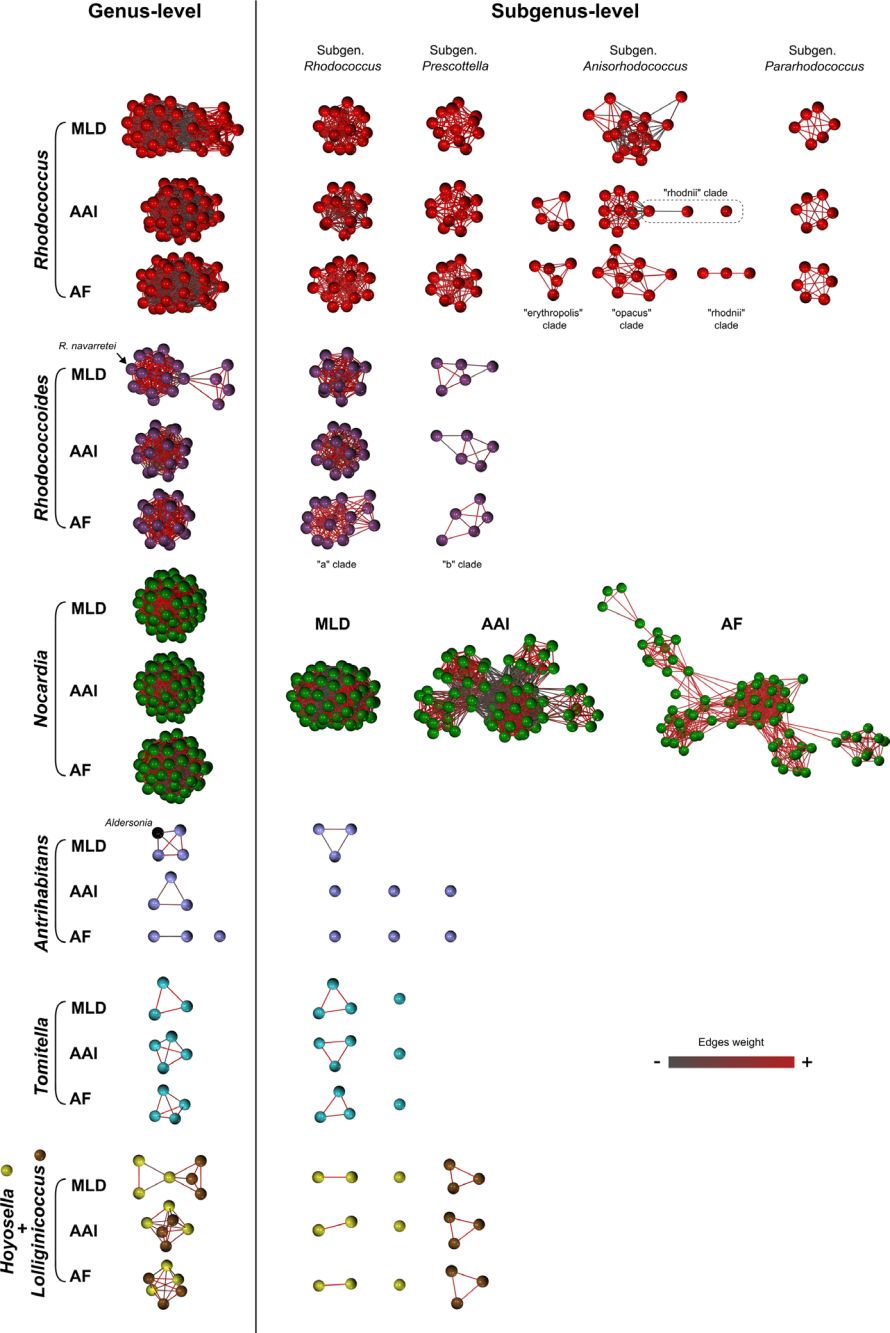
Phylogenomic 3D network analysis based on MLD and GRI (AAI, AF) matrices using the Fig. 1 dataset. Each node represents a genome, and interconnecting edges show relationships between nodes above the correlation/clustering threshold (ct). Edge colour indicates similarity strength, from grey (weaker) to red (stronger). Closely related genomes/species appear closer together in the network, with edge interconnections remaining more stable as the ct increases. Left panels, genus-level partitions; ct values: MLD 0.670, AAI 0.410 and AF 0.525. Right panels, subgenus-level partitions; ct values: MLD 0.825, AAI 0.720 and AF 0.810. The ct thresholds are set based on the taxonomic context and taxonomic level being analysed; the specific ct values for genus- or subgenus-level partitions vary depending on the genome dataset used. Note in the genus-level graphs that *R. navarretei* Carrasco *et al*. 2024 [59] is embedded in the *Rhodococcoides* network, supporting its inclusion in this genus (see also Fig. 1 legend). Also note that the monotypic genus *Aldersonia* remains connected to the *Antrihabitans* subnetwork in the MLD-based genus graphs. Previous taxonomic network analyses showed that *Aldersonia kunmingensis* DSM 45001^T^ and *Skermania pinensis* DSM 43998^T^ (also a monotypic genus) occupy an intermediate, bridging position between the *Rhodococcus* and *Nocardia* clusters, with *Aldersonia* linking to both *Antrihabitans* and *Skermania* [11]. This relationship is also apparent in Fig. S2, where a lower ct was applied to the **Fig. 2 **dataset, allowing visualization of supragenus-level relationships.

As observed in our previous *Mycobacteriales* study [11], a specific range of MLD/GRI ct cutoffs recapitulated the genus structure of the taxonomic context under analysis, isolating the genera as discrete subnetworks (Fig. 2, left). An exception was the *Lolliginicoccus* circumscription, which was fully embedded within the *Hoyosella* genus subnetwork in all three genus-level MLD/GRI network graphs. This result was consistent with the tree clustering data (Fig. 1) and with AAI scores to *Hoyosella* genomes above the ≤65% standard threshold for genus delimitation [17,20] (mean value, 69.26%), further supporting the conclusion that the *Lolliginicoccus* genus is not taxonomically justified.

By increasing the ct cutoffs to isolate the *Prescottella* sublineage circumscription in a self-contained subnetwork, additional discrete subnetworks (putative subgenera) were formed which corresponded to each of the previously identified rhodococcal sublineages (Fig. 2, right). The same was observed with the two main *Rhodococcoides* sublineages (ref. [11] and herein), while all the species of the genus *Nocardia* remained connected in a single subnetwork (Fig. 2, right), closely mirroring the ML tree clustering partitions (Fig. 1).

Only in one instance, namely, the AAI/AF-based network graphs of *Rhodococcus* sublineage no. 3+4, did the subgenus-level partitions split the corresponding circumscription into more than one subnetwork (Fig. 2). These 3+4 ‘subgeneric’ subnetworks correspond to the main internal branches of this sublineage (see Figs 1 and S1), which show differences in genome size and topology: the ‘erythropolis’ clade has circular genomes of ≈6 Mbp; the ‘opacus/jostii’ clade has large linear genomes of >8–9 Mpb; and the ‘rhodnii’ clade has smaller circular genomes of 4–5 Mbp. This internal genomic heterogeneity is unique among the rhodococcal lineages and may explain the observed fragmentation of sublineage 3+4 in the AAI/AF-based subgenus-level network graphs.

## Proposal to lower the genus *Prescottella* Sangal *et al*. 2022 to subgenus rank

**Fig. 3.**
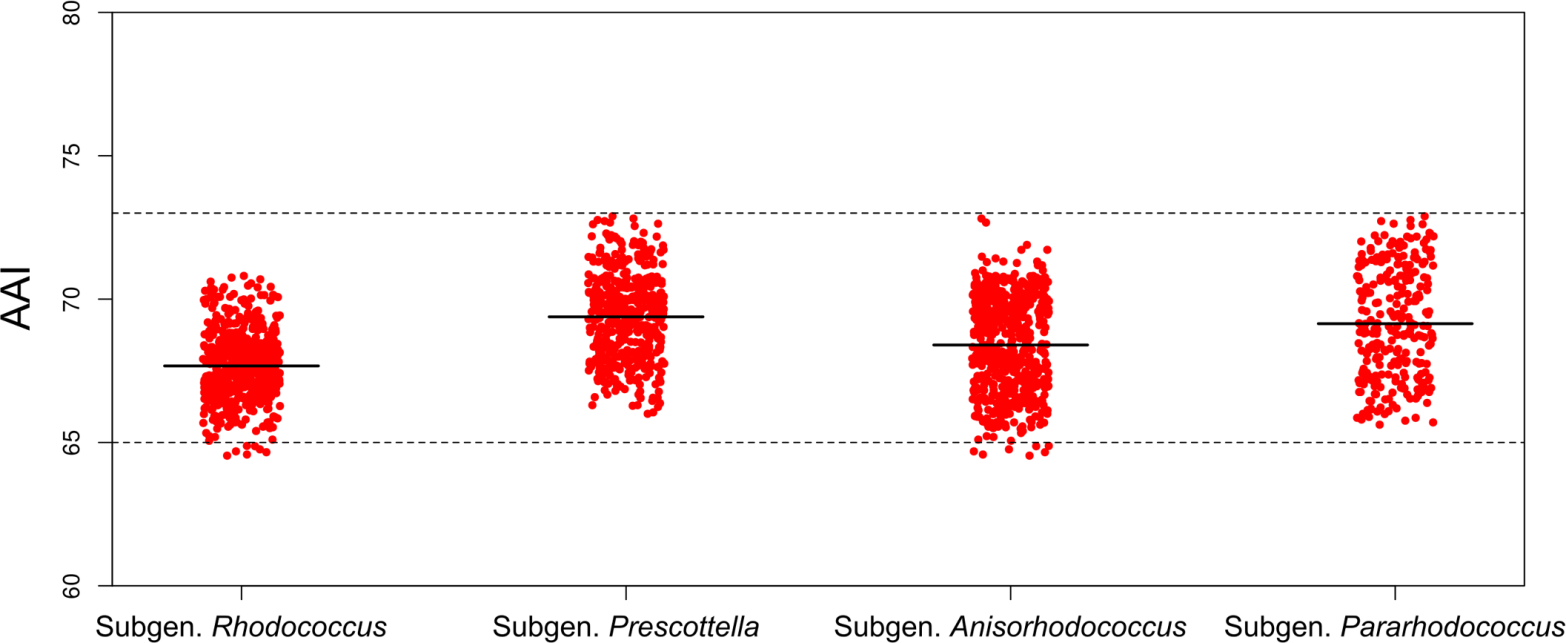
Scatter plots of *Rhodococcus* inter-subgenus AAI scores. The horizontal dashed line indicates the standard 65% AAI threshold for genus demarcation [17,18, 20]. The “upper” rhodococcal subgenus demarcation boundary is AAI=73%. The average AAI value for each subgenus is represented by a black horizontal line.

To address the problematic coexistence of two different names for the same pathogen *– R. equi* and its later homotypic synonym *P. equi* – we propose, based on our phylogenomic analyses, to reclassify the genus *Prescottella* Sangal *et al*. 2022 [15] as a subgenus of the genus *Rhodococcus* Zopf 1891 (Approved Lists 1980) emend. Val-Calvo and Vázquez-Boland 2023 [40]. For internal taxonomic consistency, we further propose the other main rhodococcal monophyletic sublineages – identified in this work and previous phylogenomic studies [11,45] – as subgenera within the genus *Rhodococcus*. All *Rhodococcus* subtaxa defined in this study exhibit average inter-subgeneric AAI values comprised between 65% (the genus demarcation standard) and 73% (Fig. 3).

Our approach to subgenus definition is conservative in that we selected a taxon demarcation threshold within the *Prescottella*-level range that minimizes the creation of new names. Accordingly, the *Rhodococcus* monophyletic sublineage 3+4, which includes three distinct lines of descent within the *Prescottella*-level subgenus definition range (i.e. the ‘erythropolis’, ‘opacus/jostii’ and ‘rhodnii’ clades) (Figs 1 and S1), was treated as a single subgenus. Because this taxon comprises species of varying genome sizes and topologies (see above), we propose the subgenus name *Anisorhodococcus*. The classification of species with different genome sizes within the same genus – or, as in this case, subgenus – is entirely acceptable. Although genome size is linked to bacterial phylogeny at a broader scale [46], it may vary within specific groups of closely related bacteria due to niche-adaptive gene content expansion or contraction. Additionally, genome size influences genome topology; specifically, larger actinomycetal genomes tend to be linear [47]. Therefore, genome size lacks strict taxonomic value [48,50].

The *Rhodococcus* subgenus nomenclature was proposed according to the precepts of the ICNP [1], as follows. Rule 49 states that ‘when a genus is lowered in rank to subgenus, the original name must be retained unless it is rejected under the Rules’. Rule 39a states that ‘If a genus is divided into two or more genera or subgenera, the generic name must be retained for one of these’. Finally, Rule 39b states that ‘When a particular species has been designated as the type, the generic name must be retained for the genus which includes that species’. Accordingly, the name *Prescottella* is used here to designate the subgenus corresponding to sublineage 2, which contains *R. equi*, while *Rhodococcus* is retained as the name of sublineage 1, which contains the type species of the genus, *Rhodococcus rhodochrous*. To minimize subjectivity in the rank demarcations, we used the tree clustering and MLD/GRI-based network analysis approach previously applied to reassess the *Mycobacteriales* taxonomy [11]. Using these criteria, the two sublineages within the genus *Rhodococcoides* Val-Calvo and Vázquez-Boland 2023 would also warrant subgenus status. However, we do not propose the creation of new subgenera for these sublineages, as our focus here is restricted to *Rhodococcus* ‘*sensu stricto*’ (i.e. as recently emended by Val-Calvo and Vázquez-Boland) [11,40]. Our main aim is addressing the confusion – and taxonomic implications – arising from the proposal of the nested genus *Prescottella* within the rhodococcal monophyletic radiation.

## Application to the split of *Mycobacterium*

Another obvious candidate for applying the subgenus strategy outlined here is the subdivision of the monophyletic genus *Mycobacterium* Lehmann and Neumann 1896 into five separate genera, proposed by Gupta *et al*. [6]. The new genus names resulting from Gupta *et al*.’s taxonomic revision are currently used by databases but are largely rejected by the end-user community, specifically the mycobacteriologists [12,14,31].

**Fig. 4.**
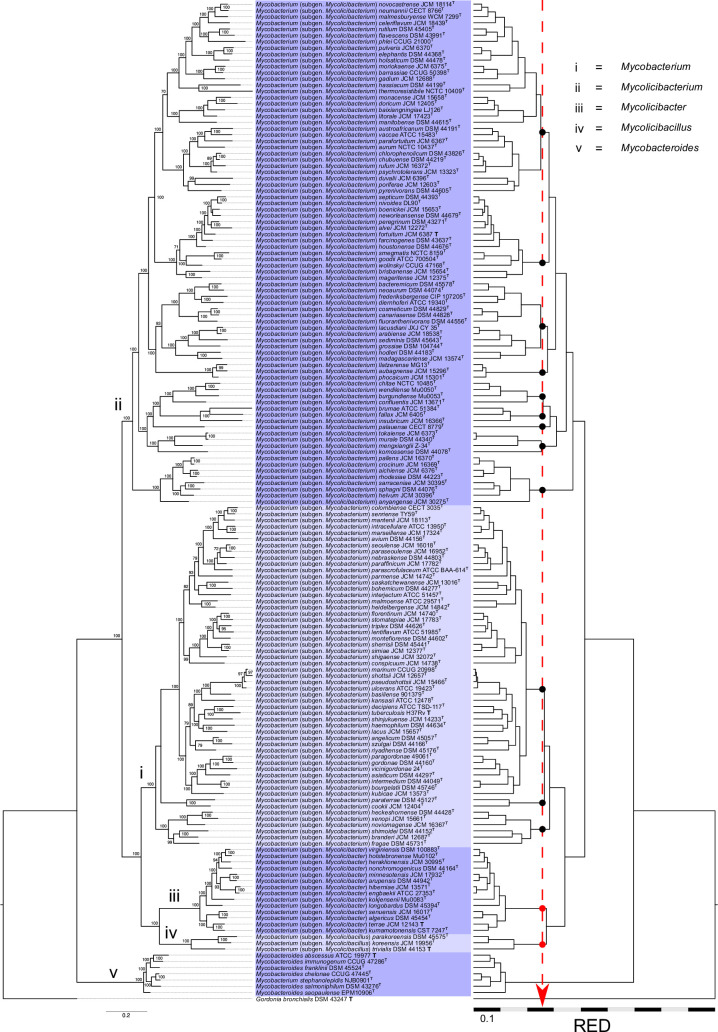
Mycobacterial whole-genome ML phylogeny. Left, non-normalized tree; right, RED-normalized tree. Gordonia bronchialis DSM 43247T was used as an outgroup (see Supplementary Dataset, sheet 1). The tree was inferred from a concatenated alignment of universal protein markers using PhyloPhlAn [67] and constructed with IQtree v2.0.7 [68] under the LG+F+R8 substitution model. The ML tree is fully consistent with previously published mycobacterial phylogenies [6,11, 12]. Mycobacterial species labels are enclosed in boxes shaded in alternating dark and light blue, corresponding to the five-genus partitions proposed by Gupta et al. [6], which are treated as subgenera in this study (with the exception of Mycobacteroides; see text). The RED-normalized tree was partitioned using a taxonomic context-uniform TreeCluster [38] threshold allowing the separation of the more distal Mycolicibacter and Mycolicibacillus taxa as independent clusters (red vertical dashed arrow). Note that applying this clustering cutoff leads to substantial fragmentation of the other genera proposed by Gupta et al. [6]; see text for details. Scale bar, amino acid substitutions per site. UltraFast Bootstrap support values (1,000 replicates) are shown. Tree visualized using FigTree v1.4.4 (http://tree.bio.ed.ac.uk/software/figtree/). The genomes used for each of the listed species are from the type strains (indicated by superscript T, a bold T in normal font designates genus/subgenus type species); their accession numbers are provided in the Supplementary Dataset table.

As a first step, we assessed the taxonomic consistency of Gupta *et al.*’s [6] *Mycobacterium* partitions by mapping the five proposed genera onto an ML tree constructed using 160 mycobacterial genomes (Fig. 4, left). The tree shows that, apart from an early diverging clade (sublineage ‘v’, assigned to the genus *Mycobacteroides*), the majority of the *Mycobacterium* (*sensu lato*) circumscription diverges into two major branches. One of these branches was subdivided by Gupta *et al*. into three unequal genus partitions. The first corresponds to the larger of the two sister clades that form this branch (labelled sublineage ‘i’ in the tree; Fig. 4, left), which contains the type species *Mycobacterium tuberculosis* and was assigned to an emended genus *Mycobacterium* [6]. The smaller sister clade was further divided into two novel genera: *Mycolicibacter* (sublineage ‘iii’) and *Mycolicibacillus* (sublineage ‘iv’) (Fig. 4, left). The second major branch (labelled sublineage ‘ii’) was assigned by Gupta *et al*. [6] to the genus *Mycolicibacterium* (Fig. 4, left). This analysis clearly shows that the genus partitions proposed by Gupta *et al*. [6] are not taxonomically consistent in terms of rank and position in the mycobacterial tree hierarchy.

The lack of consistency of the mycobacterial five-genus split is most evident in a RED-normalized ML tree (Fig. 4, right). When applying a context-uniform partitioning [11] that preserves the more distal *Mycolicibacter* and *Mycolicibacillus* demarcations, the mycobacterial radiation is subdivided into a total of 14 taxa (subgenera) of equivalent rank (Fig. 4, right). Alternatively, if *Prescottella* is taken as the subgenus partitioning reference across the broader *Mycobacteriales* taxonomic context [11] (Fig. S3), *Mycobacterium* (*sensu lato*) is subdivided into three subgenera (labelled A, B, C), corresponding to Gupta *et al.*’s [6]: (A) *Mycobacterium* (emend.)+*Mycolicibacter+Mycolicibacillus*; (B) *Mycolicibacterium*; and (C) *Mycobacteroides*.

Given that the five-genus split cannot be reconstituted using a context-uniform demarcation approach, applying our taxon delineation methodology is unwarranted in this case. We therefore propose the pragmatic solution of simply lowering the new mycobacterial genera proposed by Gupta *et al*. [6] to the subgenus category. This would allow the entire mycobacterial radiation (with the exception of the *Mycobacteroides* circumscription, see below) to be designated again under the same generic name *Mycobacterium*. In addition, each subgenus would retain the ‘generic’ names proposed by Gupta *et al*. [6] and currently used in databases, thus enabling users to easily recognize the relationship between the two nomenclatures at a glance.

The *Mycobacteroides* clade (mycobacterial sublineage ‘v’; Fig. 4) requires special consideration. Our recent taxonomic analysis of the *Mycobacteriales* found that, in contrast to the other mycobacterial genera proposed by Gupta *et al*. [6], the early-diverging mycobacterial clade ‘v’ containing *Mycobacteroides abscessus* ATCC 19977^T^ does warrant independent genus status [11]. Using a context-uniform demarcation approach to ensure taxonomic consistency across the *Mycobacteriales*, the *Mycobacteroides* clade consistently emerged as a distinct taxon, equivalent in rank to other established genera within the order’s circumscription. This includes, for example, *Gordonia*, *Williamsia*, *Tsukamurella* and *Hoyosella*. The separation of *Mycobacteroide*s as an independent genus is further supported by AAI scores ≤65% when compared with other mycobacterial clades and *Mycobacteriales* genera. In contrast, the taxon discreteness threshold for delineating the four proposed *Mycobacterium* subgenera is ≤73%, consistent with the values for the rhodococcal subgenera (see above and Fig. S4). The possibility of recognizing *Mycobacteroides* as an independent genus was also suggested in the recent taxonomic study by Meehan *et al*. in which the reconstitution of the genus *Mycobacterium* was proposed [14]. Nevertheless, the mycobacterial researchers appear to prefer including the *Mycobacteroides* circumscription within a single, unified genus, *Mycobacterium* [13]. For the sake of consistent genus demarcation across the *Mycobacteriales*, we do not propose reclassifying *Mycobacteroides* Gupta *et al*. 2018 emend. Val-Calvo and Vázquez-Boland 2023 as a subgenus of *Mycobacterium*; we leave this to the discretion of the mycobacteriologists.

## Taxonomic conclusions

The strategy of reducing the rank of new genera created by ‘genus-oversplitting’ to the subgenus category offers, in our opinion, a reasonable ‘compromise’ solution to the conundrum posed by the ‘preference for latest validly published name’ database policy. This practice potentially fixes, *ad perpetuum*, the most recently proposed name in databases, unless replaced by new validly published names – in this case, new subgeneric taxa, as proposed here.

Although the use of the subgenus category was discouraged in an earlier publication by the Judicial Commission of the ICSP [51], this body is not supposed to rule on taxonomy, and the subgenus remains an option under the Prokaryotic Code. As a taxonomic category supported by the ICNP, its application falls within the scope of freedom of taxonomic thought [1]. We believe that, for the specific purpose outlined here, the use of the subgenus rank is acceptable, justified and useful. Interestingly, a review of the literature revealed that the same solution was recently proposed to resolve equivalent issues in zoological taxonomy [52]. In prokaryotic taxonomy, adopting this approach could help mitigate the confusion and nomenclatural instability caused by recent subdivisions of monophyletic genera and ensuing reclassification of many species under new genus designations. It also restores a key aspect of binomial nomenclature: the genus name’s informative value in indicating close phylogenetic and biological relatedness among the organisms it designates. This is lost when new generic names are created after a genus split.

Another useful application of subgenus designations is to provide a formal means to label and differentiate major intra-generic clades in large and diverse genera. In *Rhodococcus*, for example, these clades are currently designated by numbers [11,45, 53, 54] or by the epithet/name of the prototype species (see above) [55,57], underscoring the need for regulated nomenclature – one that subgeneric names would appropriately satisfy. The same applies to mycobacterial sublineages (subgenera).

It could be argued that creating new subgenera potentially contradicts Principle 1(3) of the Prokaryotic Code, which advises against ‘the useless creation of names’ [1]. However, names are not useless under the Code if they have a taxonomic purpose that is not already fulfilled by a validly published and legitimate name [26]. Moreover, since the subgenus is an optional taxonomic category (Rule 5b of the Code) [1], the proposed subgeneric names are unlikely to be used frequently in practice. The prokaryotes concerned will mostly continue to be referred to by their generic names (in our examples, *Rhodococcus* and *Mycobacterium*), an outcome explicitly intended by our proposal.

Other taxonomic conclusions from this study include two reclassifications.

One concerns the recently proposed genus *Lolliginicoccus* Miyanishi *et al.* 2023 [34], which is not supported by our analyses – indeed, it appears to represent another example of the recent genus over-splitting trend. Consequently, of the three currently recognized *Lolliginicoccus* species, two should revert, in our opinion, to their basionyms/homotypic synonyms *Hoyosella lacisalsi* Yang *et al*. 2021 and *Hoyosella suaedae* Liu *et al*. 2021. The third species, *Lolliginicoccus levis* Miyanishi *et al*. 2023, should be reclassified as a new combination in *Hoyosella*.

The second reclassification concerns the recently described species *R. navarretei*. Our ML phylogeny and taxonomic network analyses unambiguously place this species within the *Rhodococcoides* radiation. We therefore propose the new combination *Rhodococcoides navarretei* comb. nov.

## Final remarks

A preprint version of this manuscript, posted on 27 April 2025 [58], was presented and discussed at the recently established ICSP Ad Hoc Committee on Mitigating Changes in Prokaryotic Taxonomy (CoMiCProN). The approach proposed in this study to address nomenclatural confusion and instability resulting from monophyletic genus over-splitting was endorsed by CoMiCProN. The use of the underutilized subgenus category is recommended for this purpose. CoMiCProN stresses that subgenera should be applied only in this specific context and does not advocate or support their use in other circumstances [59].

## Taxonomic descriptions

A comprehensive list of all genus name changes resulting from the creation of the rhodococcal and mycobacterial subgenera reported here can be found in the preprint version of this article [58].

## New subgenera

### *Rhodococcus* (Zopf 1891) subgen. nov.

(Rho.do.coc’cus. Gr. neut. n. *rhodon*, the rose; N.L. masc. n. *coccus*, coccus; from Gr. masc. n. *kokkos*, grain, seed; N.L. masc. n. *Rhodococcus*, a red coccus).

Type subgenus of the genus *Rhodococcus* Zopf 1891 (Approved Lists 1980) emend. Val-Calvo and Vázquez-Boland 2023. The proposed subgenus *Rhodococcus* would be automatically formed in the application of ICNP Rule 39a upon creation of the *Rhodococcus* subgenera described here. The *Rhodococcus* (subgen. *Rhodococcus*) circumscription is comprised of the species *R. aeterivorans*, *R. artemisiae*, *R. chondri*, *R. coprophilus*, *R. gordoniae*, *R. indonesiensis*, *R. phenolicus*, *R. pyridinivorans*, *R. rhodochrous*, *R. ruber*, *R. yananensis* and *R. zopfii*.

The type species of the subgenus *Rhodococcus* is *R. rhodochrous* (Zopf 1891) Tsukamura 1974 (Approved Lists 1980).

### *Anisorhodococcus* subgen. nov.

(An.i.so.rho.do.coc’cus*.* Gr. masc. adj. *anisos*, unequal, dissimilar; N.L. masc. n. *Rhodococcus*, a genus name; N.L. masc. n. *Anisorhodococcus*, unequal, dissimilar or uneven *Rhodococcus*, a subgenus of the genus *Rhodococcus*).

Subgenus of genus *Rhodococcus* Zopf 1891 (Approved Lists 1980) emend. Val-Calvo and Vázquez-Boland 2023. Members have the same general morphological, physiological and chemotaxonomic characteristics of the genus *Rhodococcus* as described by Goodfellow and Alderson [60] and by Jones and Goodfellow [55]. Contains the species *Rhodococcus* (subgen. *Anisorhodococcus*) *erythropolis*, *R. globerulus*, *R. jostii*, *R. koreensis*, *R. pseudokoreensis*, *R. opacus*, *R. wratislaviensis*, *R. marinonascens*, *R. oxybenzonivorans*, *R. rhodnii* and *R. triatomae*. They originate from diverse environments, including soil, rhizosphere ecosystem, marine sediments and xenobiotic-contaminated sites, or are found as part of the gut microbiota of triatomine hematophagous *Hemiptera* (kissing bugs) in the *Reduviidae* family. The species of this subgenus are characterized by genomes of variable size and topology. The phenolic compound degraders *Rhodococcus jostii* and *Rhodococcus koreensis* have large genomes ≈10 Mbp in size, likely as a result of catabolic gene network amplification, whereas the insect gut symbionts *Rhodococcus rhodnii* and *Rhodococcus triatomae* have smaller genomes of 4.5 to 4.7 Mbp, presumably due to host-adaptive reductive evolution. The species with larger genomes have linear chromosomes and several extrachromosomal elements, from large (0.4 to 1 Mbp) invertron-like linear replicons to smaller circular plasmids; those with intermediate (e.g. *R. marinonascens*, 4.9 Mbp) and smaller (*R. rhodnii* and * R. triatomae*) genomes have circular chromosomes. G+C contents range between 64.5 and 69.5 mol%. Subgenus *Anisorhodococcus* forms a large monophyletic sublineage with three distinct lines of descent and can be distinguished from other *Rhodococcus* subgenera relative evolutionary divergence (RED)-normalized tree clustering and network analysis of MLD matrices, where they form an independent cluster or subnetwork when applying clustering cutoffs that isolate the *Prescottella* sublineage/subgenus as an independent cluster/subnetwork. In pairwise comparisons, the inter-subgenus AAI scores of the *Rhodococcus* (subgen. *Anisorhodococcus*) circumscription are ≤73%.

The type species of the subgenus *Anisorhodococcus* is *Rhodococcus erythropolis* (Gray and Thornton 1928) Goodfellow and Alderson 1979 (Approved Lists 1980).

### *Pararhodococcus* subgen. nov.

(Pa.ra.rho.do.coc’cus. Gr. prep. *para*, beside, next to; N.L. masc. n. *Rhodococcus*, a genus name; N.L. masc. n. *Pararhodococcus*, near *Rhodococcus*).

Subgenus of the genus *Rhodococcus* Zopf 1891 (Approved Lists 1980) emend. Val-Calvo and Vázquez-Boland 2023. Members share the general morphological, physiological and chemotaxonomic characteristics of the genus *Rhodococcus* as described by Goodfellow and Alderson [60] and by Jones and Goodfellow [55]. Contains the species *Rhodococcus* (subgen. *Pararhodococcus*) *maanshanensis*, *R. olei*, *R. oryzae*, *R. spelaei* and *R. tukisamuensis*, isolated from soil in different ecosystems (mountainous, agricultural, cavern and urban). All have a genomic G+C content of 69–70 mol% and a size between 4.8 and 6.2 Mbp. They form the earliest-diverging branch of the monophyletic genus *Rhodococcus* Zopf 1891 (Approved Lists 1980) emend. Val-Calvo and Vázquez-Boland 2023 in a core-genome phylogenetic tree. They can be differentiated from other rhodococci by means of relative evolutionary divergence (RED)-normalized tree clustering and GRI-based network analysis, where they form an independent cluster or subnetwork when applying clustering or graph cutoffs that isolate the *Prescottella* sublineage/subgenus as an independent cluster/subnetwork. In pairwise comparisons, the inter-subgenus AAI score of the current *Rhodococcus* (subgen. *Pararhodococcus*) circumscription is ≤73%.

The type species of the subgenus *Pararhodococcus* is *Rhodococcus maanshanensis* Zhang *et al.* 2002.

### *Prescottella* (Sangal *et al*. 2022) subgen. nov.

(Pres.cot.tel’la. N.L. fem. dim. n. *Prescottella*, in honour of John Prescott for his pioneering research contributions into the pathogenicity and epidemiology of *R. equi*).

The description of this taxon is as given by Sangal *et al*. [15]. Previously described as a genus, it is here lowered in rank to a subgenus of the genus *Rhodococcus* Zopf 1891 (Approved Lists 1980) emend. Val-Calvo and Vázquez-Boland 2023. Core-genome phylogenies, as well as RED-normalized tree clustering and GRI- and ML distance-based network analyses, all show that the *Rhodococcus* (subgen. *Prescottella*) species belong to an internal lineage of the genus *Rhodococcus*. This sublineage is defined by an intra-subgenus average AAI score of 83.6% and inter-subgenus AAI scores of ≤73%. It contains the species *Rhodococcus* (subgen. *Prescottella*) *agglutinans*, *R. defluvii*, *R. equi*, *R. parequi*, *R. soli*, *R. spongiicola*, *R. subtropicus* and *R. xishaensis.*

The type species of the subgenus *Prescottella* is *R. equi* (Magnusson 1923) Goodfellow and Alderson 1977 (Approved Lists 1980).

### *Mycobacterium* (Lehmann and Neumann 1896) subgen. nov.

(My.co.bac.te’ri.um. Gr. masc. n. *mykês*, a mushroom, fungus; N.L. neut. n. *bacterium*, a rod; N.L. neut. n. *Mycobacterium*, a fungus rodlet).

Type subgenus of the genus *Mycobacterium* Lehmann and Neumann 1896 (Approved Lists 1980) emend. Val-Calvo and Vázquez-Boland 2023. It would also be automatically formed in application of ICNP Rule 39a upon creation of the other *Mycobacterium* subgenera described here.

The type species of the subgenus *Mycobacterium* is *M. tuberculosis* (Zopf 1883) Lehmann and Neumann 1896 (Approved Lists 1980).

### *Mycolicibacillus* (Gupta *et al*. 2018) subgen. nov.

(my.co.li.ci.ba.cil’lus. n.l. neut. n. *acidum mycolicum*, mycolic acid; l. masc. n. *bacillus*, a small staff or rod; N.L. masc. n. *Mycolicibacillus*, a genus of mycolic acid-containing rod-shaped bacteria).

The description of this taxon is as given by Gupta *et al.* [6]. Previously described as a genus, it is here lowered in rank to subgenus of the genus *Mycobacterium* Lehmann and Neumann 1896 (Approved Lists 1980).

The type species is *Mycobacterium triviale* Kubica *et al.* 1970 (Approved Lists 1980).

### *Mycolicibacter* (Gupta *et al*. 2018) subgen. nov.

(My.co.li.ci.bac’ter. N.L. neut. n. *acidum mycolicum*, mycolic acid; N.L. masc. n. *bacter*, rod; N.L. masc. n. *Mycolicibacter*, a genus of mycolic acid-containing rod-shaped bacteria).

The description of this taxon is as given by Gupta *et al.* [6]. Previously described as a genus, it is here lowered in rank to a subgenus of the genus *Mycobacterium* Lehmann and Neumann 1896 (Approved Lists 1980). The subgenus *Mycolicibacter* includes, in addition to the circumscription of the genus *Mycolibacter* Gupta *et al*. [6], the following species recently described within the genus *Mycobacterium: M. holstebronense* Iversen *et al.* 2025 and *M. kokjensenii* Iversen *et al.* 2025 [31].

The type species is *Mycobacterium terrae* Wayne 1966 (Approved Lists 1980).

### *Mycolicibacterium* (Gupta *et al*. 2018) subgen. nov.

(My.co.li.ci.bac.te’ri.um. N.L. neut. n. *acidum mycolicum*, mycolic acid; N.L. neut. n. *bacterium*, a small rod; N.L. neut. n. *Mycolicibacterium*, a genus of mycolic acid-containing rod-shaped bacteria).

The description of this taxon is as given by Gupta *et al.* [6]. Previously described as a genus, it is here lowered in rank to a subgenus of the genus *Mycobacterium* Lehmann and Neumann 1896 (Approved Lists 1980). The subgenus *Mycolicibacterium* includes, in addition to the circumscription of the genus *Mycolicibacterium* Gupta *et al*. 2018 [6], the following species recently described within the genus *Mycobacterium*: *M. barrassiae* Adékambi *et al*. 2024 [61], *M. burgundiense* Iversen *et al*. 2025 [31], *M. manitobense* Turenne *et al*. 2003 [62], *M. neumannii* Nouioui *et al*. 2017 [63] and *M. wendilense* Iversen *et al.* 2025 [31].

The type species is *Mycobacterium fortuitum* da Costa Cruz 1938 (Approved Lists 1980).

## Emendations

### *Hoyosella* Jurado *et al.* 2009 emend.

The description of this genus is as given by Jurado *et al*. [64] and Miyanishi *et al.* [34] upon transfer of the genus *Lolliginicoccus* Miyanishi *et al*. 2023 circumscription to *Hoyosella*, of which they are an internal sub-branch based on context-uniform RED-normalized *Mycobactariales* core-genome phylogenetic tree clustering [11]. After the *Lolliginicoccus* reclassification, the *Hoyosella* circumscription comprises the species *Hoyosella altamirensis*, *H. lacisalsi*, *H. levis*, *H. rhizosphaerae*, *H. suaedae* and *H. subflava*.

The type species of the genus is *H. altamirensis* Jurado *et al*. 2009.

## New combinations

### *Rhodococcoides navarretei* (Carrasco *et al*. 2024) comb. nov.

(na.var.re’te.i. N.L. gen. masc. n. *navarretei*, named in honour of Group Commander of the Chilean Air Force Eduardo Navarrete Pizarro, chief of the Union Glacier Station during the scientific expedition that collected the soil samples from which the bacterium was isolated, who died in the 2019 Chilean military plane crash over the Drake Passage).

Basonym: *Rhodococcus navarretei* Carrasco *et al.* 2024.

The description of this taxon is as given by Carrasco *et al.* [65]. The type strain has a G+C content of 64.5 mol% and a genome with a size of ≈5.3 Mbp.

Type strain is EXRC-4A-4^T^ (=LMG 33621^T^=RGM 3539^T^).

### *Hoyosella levis* (Miyanishi *et al*. 2023) comb. nov.

(le’vis. L. fem. adj. *levis*, light).

Basonym: *Lolliginicoccus levis* Miyanishi *et al.* 2023.

The description of this taxon is as given by Miyanishi *et al.* [34]. The type strain has a G+C content of 68 mol% and a genome size of ≈3.6 Mbp. The complete genomes and 16S rRNA gene sequences of the type strain are available in the DDBJ/EMBL/GenBank databases under the accession numbers ASM2600851 and LC685063, respectively.

The type strain is Y7R2^T^ (=KCTC 49749^T^= NBRC 114883^T^).

### *Mycobacteroides* *stephanolepidis* (Fukano *et al.* 2017) comb. nov.

(ste.pha.no.le’pi.dis. N.L. gen. masc. n. *stephanolepidis*, of *Stephanolepis*, the genus of the host filefish, *Stephanolepis cirrhifer*).

Basonym: *Mycobacterium stephanolepidis* Fukano *et al*. 2017

The description of this taxon is as given by Fukano *et al*. (2017) [66]. The type strain has a G+C content of 64.0% and a genome with a size of »5.0 Mbp.

Type strain: HY188T ^T^ (=CGMCC 1.16971^T^ = JCM 33467^T^).

## Supplementary material

10.1099/ijsem.0.006917 Uncited Fig. S1.

10.1099/ijsem.0.006917 Uncited Supplementary Data Sheet 1.

## Abbreviations

AAI: average amino acid identity
AF: aligned fraction of orthologous genes
CoMiCProN: Committee on Mitigating Changes in Prokaryotic Taxonomy
ct: clustering threshold
DDBJ: DNA Data Bank of Japan
+F: empirical base frequencies
GRI: genomic relatedness index
GTR: General Time-Reversible with empirical base frequencies
ICNP: International Code of Nomenclature of Prokaryotes
ICSP: International Committee on Systematics of Prokaryotes
INSDC: International Nucleotide Sequence Database Collaboration
ML: maximum likelihood
MLD: maximum likelihood distance
NCBI: National Center for Biotechnology Information
+R: FreeRate model categories
RED: relative evolutionary divergence
