# Corrigendum: Leveraging the subgenus category to address monophyletic genus over-splitting: illustration with recently proposed Mycobacteriales genera

**DOI:** 10.1099/ijsem.0.007233

**Published:** 2026-07-08

**Authors:** Jorge Val-Calvo, Mariela Scortti, Markus Göker, José A. Vázquez-Boland

**Affiliations:** 1 Microbial Pathogenomics Laboratory, Edinburgh Medical School (Biomedical Sciences) and Institute for Regeneration and Repair (IRR), Edinburgh BioQuarter, Edinburgh EH16 4UU, Scotland, UK; 2 Leibniz Institute DSMZ, German Collection of Microorganisms and Cell Cultures, 38124 Braunschweig, Germany

In the published version of this article, an error was identified in the description of *Mycobacteroides stephanolepidis* (Fukano *et al*. 2017) comb. nov. The type strain was incorrectly listed as: HY188T^T^ (=CGMCC 1.16971^T^ = JCM 33467^T^).

The correct type strain designations are as follows:

Type strain: NJB0901ᵀ (= JCM 31611ᵀ = KCTC 39843ᵀ)

The authors apologise for any confusion or inconvenience caused.

