# Supplementary material for: Leveraging the subgenus category to address monophyletic genus over-splitting: illustration with recently proposed Mycobacteriales genera
Source: Int J Syst Evol Microbiol. 2025 Sep 19;75(9):006917. doi: 10.1099/ijsem.0.006917 (PMC12451644; doi:10.1099/ijsem.0.006917)
Supplement: Uncited Fig. S1. [file ijsem-75-06917-s001.pdf]

SUPPLEMENTAL MATERIAL

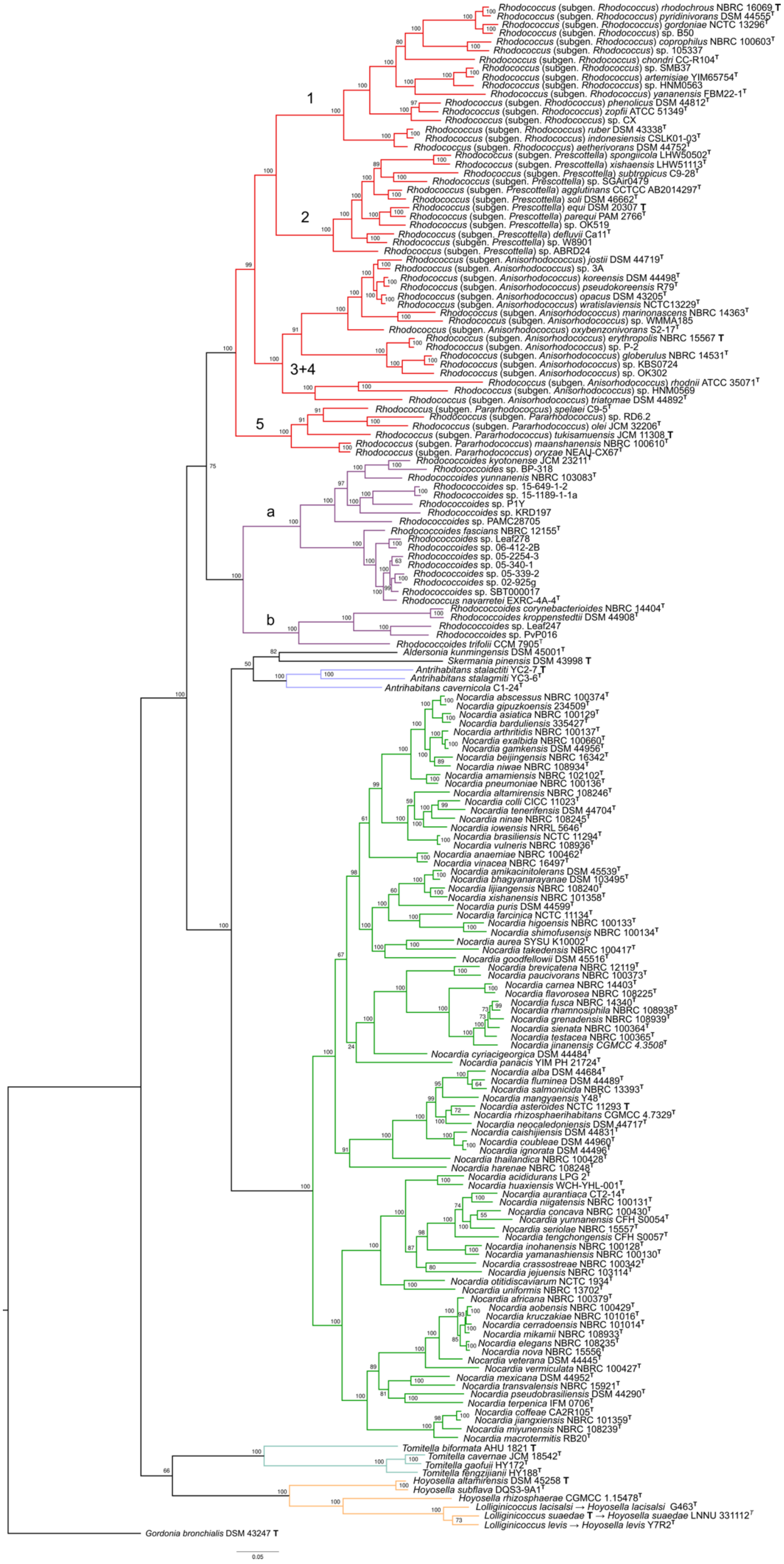

Fig. S1

Fig. S1 (cont.). Non-normalized tree of the ML phylogeny in **Fig. 1**. Based on a concatenated alignment of 65 conserved proteins selected using the Get_Homologues and Get_Phylomarkers pipelines [1, 2] from 175 *Nocardiaceae*, *Tomitellaceae* and *Hoyosellaceae* species plus *Gordonia bronchialis* DSM 43247^{T} as an outgroup. Accessions for genomes used can be found in Suppl. Dataset, sheet 1. The monotypic, single-strain taxons *Millisia brevis* NBRC 105863^{T} and *Smaragdicoccus niigatensis* DSM 44881^{T} were excluded from the phylogeny because of their uncertain position within the *Nocardiaceae* radiation, which reduced the robustness of the phylogenetic trees (long-branch attraction effect, see ref. [3]). The tree was built using Iqtree v2.0.7 [4] and the substitution model LG+F+R7. Branch colour coding and rhodoccal sugenus/sublineage labels as in **Fig. 1**. The type species of each genus are indicated by a bold-case T, for the proposed *Rhodococcus* subgenera by a T in parenthesis. UltraFastBootstraps values are shown (10,000 replicates). Scale bar, amino acid substitutions per site.Tree plotted using FigTree v1.4.4 (http://tree.bio.ed.ac.uk/software/figtree/). The genomes used for each of the listed species are from the type strains (indicated by superscript T, a bold T in normal font designates genus/subgenus type species); their accession numbers are provided in the **Supplementary Dataset** table.

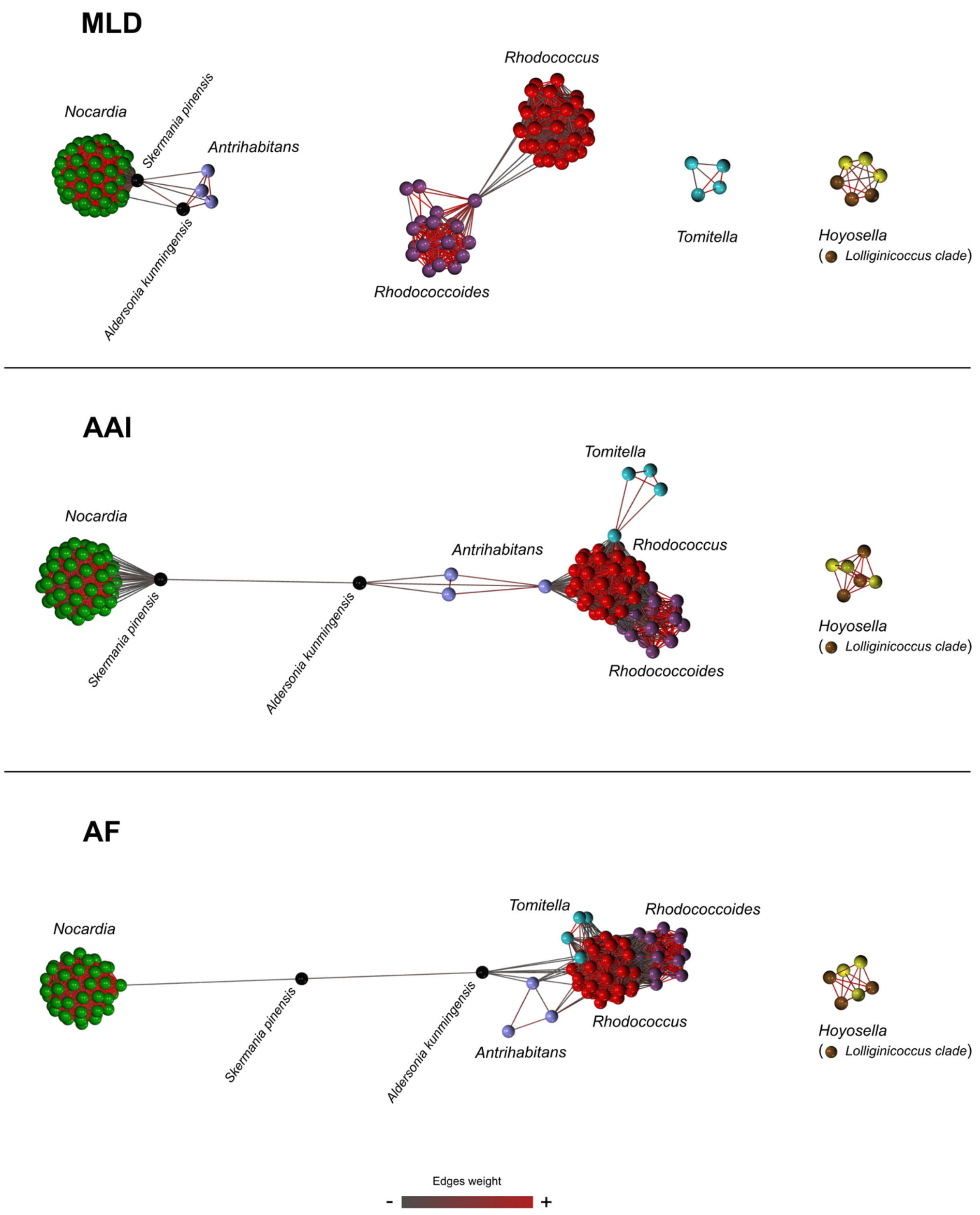

Fig. S2. Supragenus-level partitions of **Fig. 2** taxonomic network based on ML distance (MLD) and genome relatedness index (AAI and AF) pairwise comparison matrices. At the applied ct values (MLD = 0.575, AAI = 0.300, AF = 330), all three networks consistently isolate *Hoyosella* (*Hoyosellaceae* family) as an independent cluster. However, *Tomitella* (*Tomitellaceae* family) remains connected with the *Rhodococcus* subnetwork in the AAI and AF graphs (via the type species *Tomitella biformata*). The same happens with the *Antrihabitans* (Nocardiaceae family)

subnetwork. The latter connects with the *Nocardia* subnetwork through *Aldersonia kunmingensis* DSM 45001^T *and Skermania piniformis* DSM 43998^T which act as bridging nodes denoting their intermediate position between the nocardiae and the rhodococci. Scale shows edge weight from weaker (dark grey) to stronger (red).

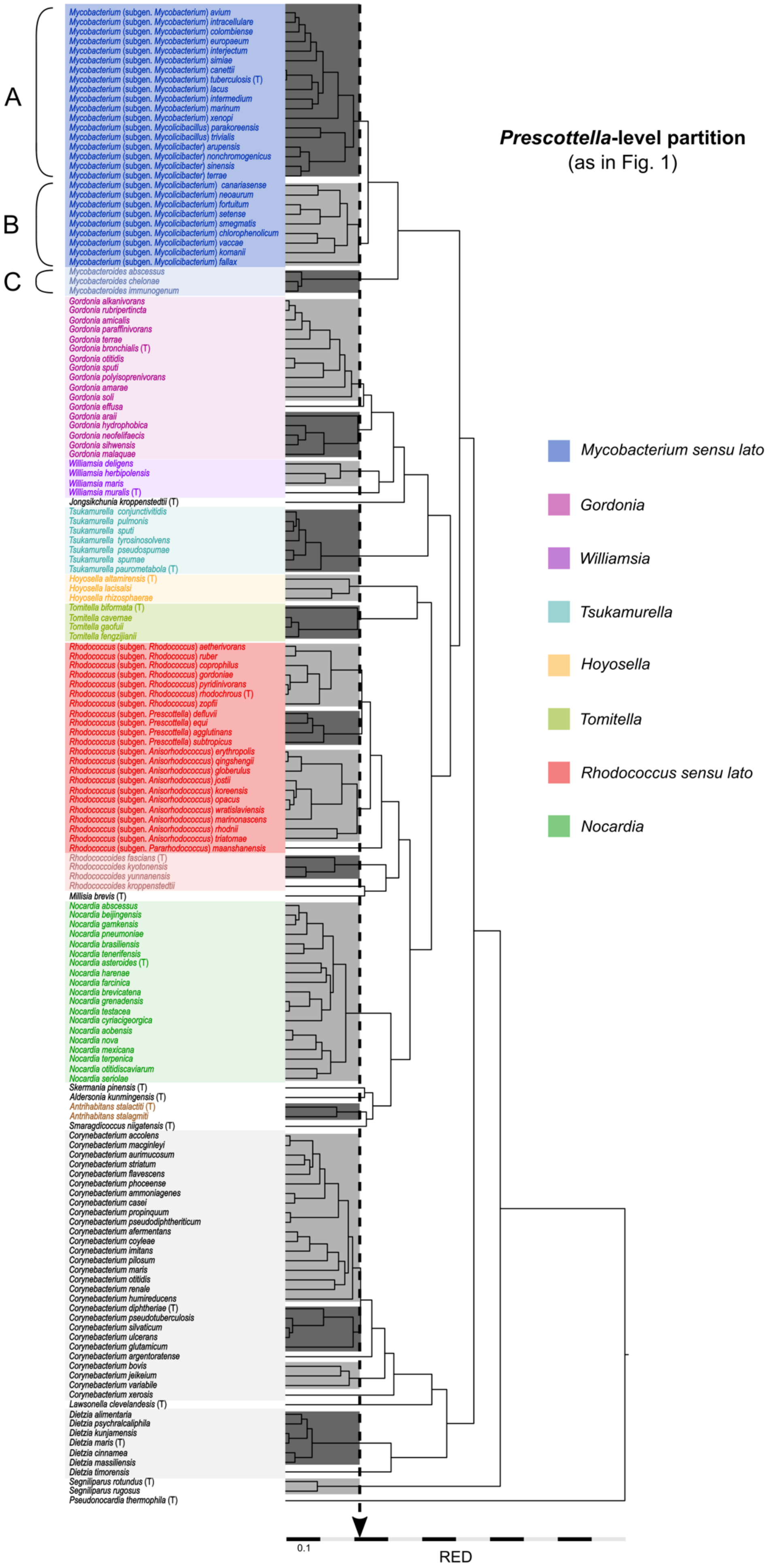

A
B
C
Mycobacterium (subgen. Mycobacterium) avium
Mycobacterium (subgen. Mycobacterium) intracellulare
Mycobacterium (subgen. Mycobacterium) colombiense
Mycobacterium (subgen. Mycobacterium) europaeum
Mycobacterium (subgen. Mycobacterium) interjectum
Mycobacterium (subgen. Mycobacterium) simiae
Mycobacterium (subgen. Mycobacterium) canettii
Mycobacterium (subgen. Mycobacterium) tuberculosis (T)
Mycobacterium (subgen. Mycobacterium) lacus
Mycobacterium (subgen. Mycobacterium) intermedium
Mycobacterium (subgen. Mycobacterium) marinum
Mycobacterium (subgen. Mycobacterium) xenopi
Mycobacterium (subgen. Mycolicibacillus) parakoreensis
Mycobacterium (subgen. Mycolicibacillus) trivialis
Mycobacterium (subgen. Mycolicibacter) arupensis
Mycobacterium (subgen. Mycolicibacter) nonchromogenicus
Mycobacterium (subgen. Mycolicibacter) sinensis
Mycobacterium (subgen. Mycolicibacter) terrae
Mycobacterium (subgen. Mycolicibacterium) canariasense
Mycobacterium (subgen. Mycolicibacterium) neoaurum
Mycobacterium (subgen. Mycolicibacterium) fortuitum
Mycobacterium (subgen. Mycolicibacterium) setense
Mycobacterium (subgen. Mycolicibacterium) smegmatis
Mycobacterium (subgen. Mycolicibacterium) chlorophenolicum
Mycobacterium (subgen. Mycolicibacterium) vaccae
Mycobacterium (subgen. Mycolicibacterium) komanii
Mycobacterium (subgen. Mycolicibacterium) fallax
Mycobacteroides abscessus
Mycobacteroides chelonae
Mycobacteroides immunogenum
Gordonia alkanivorans
Gordonia rubripertincta
Gordonia amicalis
Gordonia paraffinivorans
Gordonia terrae
Gordonia bronchialis (T)
Gordonia otitidis
Gordonia sputi
Gordonia polyisoprenivorans
Gordonia amarae
Gordonia soli
Gordonia effusa
Gordonia araii
Gordonia hydrophobica
Gordonia neofelifaecis
Gordonia sihwensis
Gordonia malaquae
Williamsia deligens
Williamsia herbipolensis
Williamsia maris
Williamsia muralis (T)
Jongsikchunia kroppenstedtii (T)
Tsukamurella conjunctivitidis
Tsukamurella pulmonis
Tsukamurella sputi
Tsukamurella tyrosinosolvens
Tsukamurella pseudospumae
Tsukamurella spumae
Tsukamurella paurometabola (T)
Hoyosella altamirensis (T)
Hoyosella lacisalsi
Hoyosella rhizosphaerae
Tomitella biformata (T)
Tomitella cavernae
Tomitella gaofuii
Tomitella fengzijianii
Rhodococcus (subgen. Rhodococcus) aetherivorans
Rhodococcus (subgen. Rhodococcus) ruber
Rhodococcus (subgen. Rhodococcus) coprophilus
Rhodococcus (subgen. Rhodococcus) gordoniae
Rhodococcus (subgen. Rhodococcus) pyridinivorans
Rhodococcus (subgen. Rhodococcus) rhodochrous (T)
Rhodococcus (subgen. Rhodococcus) zopfii
Rhodococcus (subgen. Prescottella) defluvii
Rhodococcus (subgen. Prescottella) equi
Rhodococcus (subgen. Prescottella) agglutinans
Rhodococcus (subgen. Prescottella) subtropicus
Rhodococcus (subgen. Anisorhodococcus) erythropolis
Rhodococcus (subgen. Anisorhodococcus) qingshengii
Rhodococcus (subgen. Anisorhodococcus) globerulus
Rhodococcus (subgen. Anisorhodococcus) jostii
Rhodococcus (subgen. Anisorhodococcus) koreensis
Rhodococcus (subgen. Anisorhodococcus) opacus
Rhodococcus (subgen. Anisorhodococcus) wratislaviensis
Rhodococcus (subgen. Anisorhodococcus) marinonascens
Rhodococcus (subgen. Anisorhodococcus) rhodnii
Rhodococcus (subgen. Anisorhodococcus) triatomae
Rhodococcus (subgen. Pararhodococcus) maanshanensis
Rhodococcoides fascians (T)
Rhodococcoides kyotonensis
Rhodococcoides yunnanensis
Rhodococcoides kroppenstedtii
Millisia brevis (T)
Nocardia abscessus
Nocardia beijingensis
Nocardia gamkensis
Nocardia pneumoniae
Nocardia brasiliensis
Nocardia tenerifensis
Nocardia asteroides (T)
Nocardia harenae
Nocardia farcinica
Nocardia brevicatena
Nocardia grenadensis
Nocardia testacea
Nocardia cyriacigeorgica
Nocardia aobensis
Nocardia nova
Nocardia mexicana
Nocardia terpenica
Nocardia otitidiscaviarum
Nocardia seriolae
Skermania pinensis (T)
Aldersonia kunmingensis (T)
Antrihabitans stalactiti (T)
Antrihabitans stalagmiti
Smaragdicoccus niigatensis (T)
Corynebacterium accolens
Corynebacterium macginleyi
Corynebacterium aurimucosum
Corynebacterium striatum
Corynebacterium flavescens
Corynebacterium phoceense
Corynebacterium ammoniagenes
Corynebacterium casei
Corynebacterium propinquum
Corynebacterium pseudodiphtheriticum
Corynebacterium afermentans
Corynebacterium coyleae
Corynebacterium imitans
Corynebacterium pilosum
Corynebacterium maris
Corynebacterium otitidis
Corynebacterium renale
Corynebacterium humireducens
Corynebacterium diphtheriae (T)
Corynebacterium pseudotuberculosis
Corynebacterium silvaticum
Corynebacterium ulcerans
Corynebacterium glutamicum
Corynebacterium argentoratense
Corynebacterium bovis
Corynebacterium jeikeium
Corynebacterium variabile
Corynebacterium xerosis
Lawsonella clevelandesis (T)
Dietzia alimentaria
Dietzia psychralcaliphila
Dietzia kunjamensis
Dietzia maris (T)
Dietzia cinnamea
Dietzia massiliensis
Dietzia timorensis
Segniliparus rotundus (T)
Segniliparus rugosus
Pseudonocardia thermophila (T)
0.1
RED
Prescottella-level partition
(as in Fig. 1)
Mycobacterium sensu lato
Gordonia
Williamsia
Tsukamurella
Hoyosella
Tomitella
Rhodococcus sensu lato
Nocardia

Fig. S3

Fig. S3 (cont.). Tree clustering of the *Mycobacteriales* using *Prescottella* as taxonomic context-uniform subgenus-level partitioning reference [3]. Based on the (RED)-normalized phylogenomic ML tree originally published in ref. [3]. The applied TreeCluster [5] *t* cutoff isolates the main rhodococcal sublineages/proposed *Rhodococcus* subgenera (vertical dashed arrow). This subdivides the mycobacterial tree into three main clusters which only partially correspond to Gupta *et al.* [6] taxons, as follows: (A) *Mycobacterium* + *Mycolicibacter* + *Mycolicibacillus* (B) *Mycolicibacterium*, and (C) *Mycobacteroides.* The genomes used for each of the listed species are from the type strains (except for *M. canettii* Van Soolingen *et al*. 1997, which is not validly published and has no type strain). Figure modified from **Fig. 2** of ref. [3].

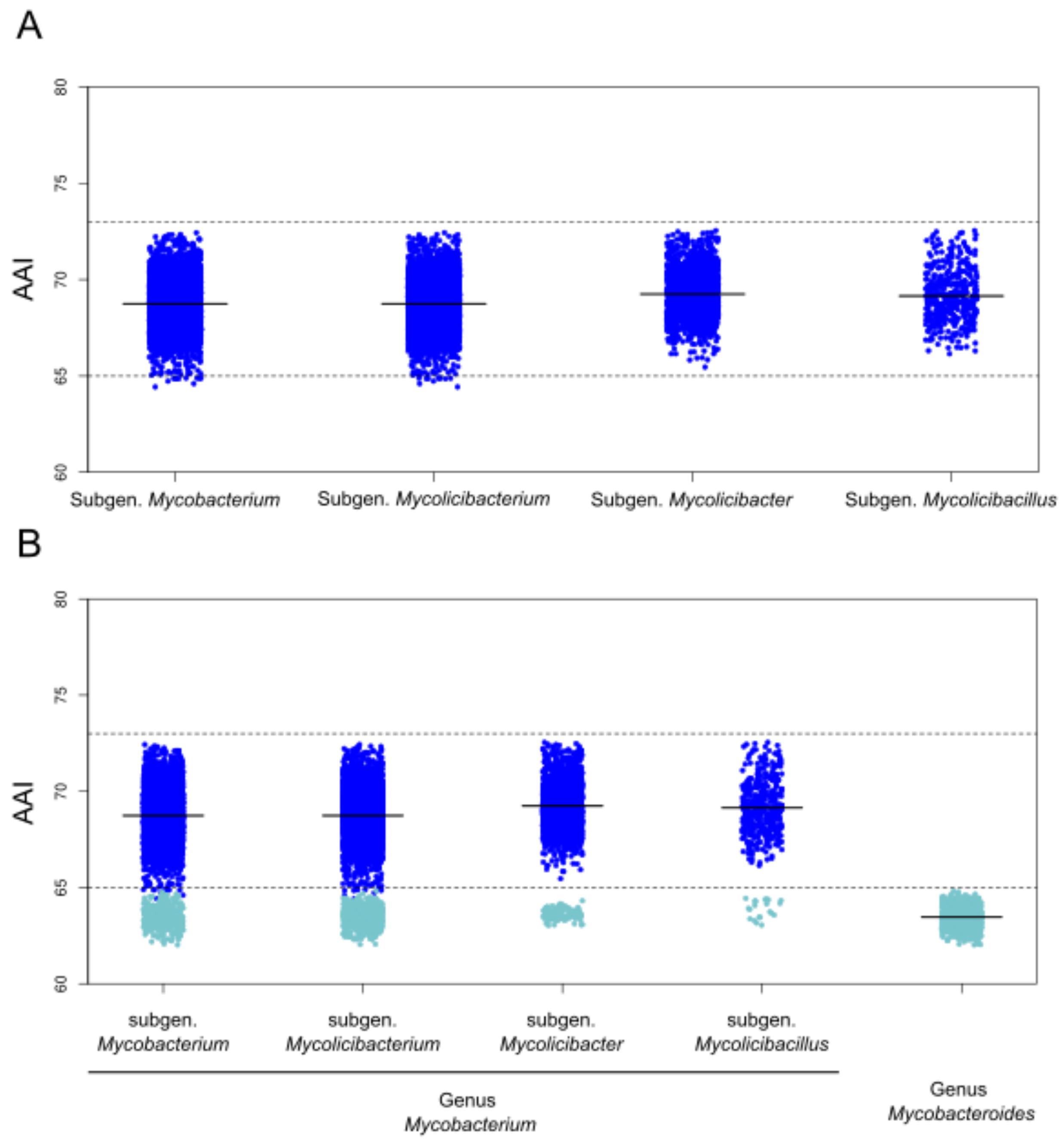

Fig. S4. AAI score demarcation of *Mycobacterium* subgenera. The average AAI values are represented by a black horizontal line. (A) scatter plots of *Mycobacterium* AAI inter-subgenus pairwise comparisons. The subgenus demarcation boundary is 73%, the same as the AAI demarcation of rhodoccal subgenera. (B) Same as in A with the AAI comparisons between the species within the four proposed *Mycobacterium* subgenera and the seven species of within the genus *Mycobacteroides* circumscription added (represented as light blue dots). Note the clear-cut separation of the latter below the 65% AAI demarcation standard for genus definition [7-9], supporting the consideration of *Mycobacteroides* as a separate genus rather than a *Mycobacterium* subgenus.

SUPPLEMENTAL REFERENCES

1. **Vinuesa P, Ochoa-Sánchez LE, Contreras-Moreira B**. GET_PHYLOMARKERS, a software package to select optimal orthologous clusters for phylogenomics and inferring pan-genome phylogenies, used for a critical geno-taxonomic revision of the genus Stenotrophomonas. *Front Microbiol* 2018;9:771. https://doi.org/10.3389/fmicb.2018.00771
2. **Contreras-Moreira B, Vinuesa P**. GET_HOMOLOGUES, a versatile software package for scalable and robust microbial pangenome analysis. *Appl Environment Microbiol* 2013;79:7696-7701. https://doi.org/10.1128/AEM.02411-13
3. **Val-Calvo J, Vazquez-Boland JA**. *Mycobacteriale*s taxonomy using network analysis-aided, context-uniform phylogenomic approach for non-subjective genus demarcation. *MBio* 2023;14:e0220723. https://doi.org/10.1128/mbio.02207-23
4. **Nguyen L-T, Schmidt HA, Von Haeseler A, Minh BQ**. IQ-TREE: a fast and effective stochastic algorithm for estimating maximum-likelihood phylogenies. *Mol Biol Evol* 2015;32:268-274. https://doi.org/10.1093/molbev/msu300
5. **Balaban M, Moshiri N, Mai U, Jia X, Mirarab S**. TreeCluster: Clustering biological sequences using phylogenetic trees. *PloS one* 2019;14:e0221068. https://doi.org/10.1371/journal.pone.0221068
6. **Gupta RS, Lo B, Son J**. Phylogenomics and comparative genomic studies robustly support division of the genus *Mycobacterium* into an emended genus *Mycobacterium* and four novel genera. *Front Microbiol* 2018;9:67. https://doi.org/10.3389/fmicb.2018.00067
7. **Konstantinidis KT, Rosselló-Móra R, Amann R**. Uncultivated microbes in need of their own taxonomy. *ISME J* 2017;11:2399-2406. https://doi.org/10.1038/ismej.2017.113
8. **Konstantinidis KT, Tiedje JM**. Prokaryotic taxonomy and phylogeny in the genomic era: advancements and challenges ahead. *Curr Opinn Microbiol* 2007;10:504-509. https://doi.org/10.1016/j.mib.2007.08.006
9. **Luo C, Rodriguez-r LM, Konstantinidis KT**. MyTaxa: an advanced taxonomic classifier for genomic and metagenomic sequences. *Nucl Acids Res*2014;42:e73-e73. https://doi.org/10.1093/nar/gku169
